# Symptoms of urinary incontinence and pelvic organ prolapse and physical performance in middle-aged women from Northeast Brazil: a cross-sectional study

**DOI:** 10.1186/s12905-019-0786-2

**Published:** 2019-07-11

**Authors:** Mariana Carmem Apolinário Vieira, Saionara Maria Aires da Câmara, Mayle Andrade Moreira, Catherine McLean Pirkle, Afshin Vafaei, Álvaro Campos Cavalcanti Maciel

**Affiliations:** 1 Physiotherapy Department of Federal University of Rio Grande do Norte, Avenida Senador Salgado Filho, S/N Caixa Postal 1524 - Campus Universitário - Lagoa Nova, CEP, Natal, RN 59072-970 Brazil; 2 Federal University of Ceará, Fortaleza, CE Brazil; 3 University of Hawai’i at Mānoa, Honolulu, HI USA; 4 Lakehead University, Thunder Bay, Ontario Canada

**Keywords:** Aging, Epidemiology, Pelvic floor, Women’s health

## Abstract

**Background:**

Reproductive history and urogynecological disorders have been associated with limitations in physical function. However, little is known about the relationship between symptoms of urinary incontinence and pelvic organ prolapse, and physical performance. Therefore, the purpose of this study was to examine whether symptoms of urinary incontinence and pelvic organ prolapse are independently associated factors with indicators of lower physical performance in middle-aged women from Northeast Brazil.

**Methods:**

This is a cross-sectional study of 381 women between 40 to 65 years old living in Parnamirim, Northeast Brazil. Physical performance was assessed by gait speed, chair stand and standing balance tests. Urinary incontinence and pelvic organ prolapse were self-reported. Multiple linear regression analyses were performed to model the effect of self-reported urinary incontinence and pelvic organ prolapse on each physical performance measure, adjusted for covariates (age, family income, education, body mass index, parity).

**Results:**

In the analysis adjusted for confounders, women reporting urinary incontinence spent, on average, half a second longer to perform the chair stand test (β = 0.505 95% CI: 0.034: 0.976). Those reporting pelvic organ prolapse shortened the balance time with eyes open by 2.5 s on average (β = − 2.556; CI: − 4.769: − 0.343).

**Conclusions:**

Symptoms of pelvic organ prolapse and urinary incontinence are associated to worse physical performance in middle-aged women. These seemingly small changes in physical performance levels are of clinical importance, since these conditions may influence women’s physical ability, with implications for other tasks important to daily functioning and should be addressed by health policies targeting women’s health and functionality.

## Background

Degenerative processes associated with aging involve multiple body systems, including the musculoskeletal system. Detrimental changes to the musculoskeletal system gradually lead to worse physical performance and functional limitation [1]. Considering the growing number or older people globally, the burden attributed to age-related mobility decline is an increasing public health concern worldwide, including in emerging economies such as Brazil [2].

Measurement of mobility decline is not always straightforward. Physical performance is defined as a measure of lower and upper extremities’ functioning and assessment of physical performance is useful for identifying early stages of functional limitation and predicting subsequent health outcomes, such as institutionalization and mortality in older populations [3]. Objective measurement of ‘standing balance’, ‘walking speed’ and ‘time to rise from a chair’ are simple and standard approaches for assessment of physical performance [4]. They have advantages in relation to self-reported measures in regards to their validity, reproducibility, sensitivity to changes [5]. They are able to provide reliable and valid indicators for individuals’ current health status and are capable of predicting subsequent adverse health outcomes in older adults, including mortality [5].

Physical performance decline with age occurs for both sexes; however, older women suffer disproportionally compared to men at similar ages [6], suggesting the existence of biological-linked or social-related factors that affect women more strongly than men [7]. One group of factors that may explain women’s relatively greater burden of physical decline and disability has been related to reproductive history. The ill effects of high parity and early age at first childbirth on chronic diseases and poor physical performance have been reported in precedent studies of elderly and middle-aged women [8, 9]. In addition, a greater number of pregnancies seem to impair the function of muscles around the pelvic floor and hips permanently and contribute to the development of detrimental gynecological disorders [10].

The dysfunction of the pelvic floor causes a range of clinical conditions including urinary incontinence and vaginal bulging, which is a specific symptom of pelvic organ prolapse [11]. It has been documented that urinary incontinence and pelvic organ prolapse are directly associated with decreased physical activity in older women [12, 13] and possibly with mobility decline [14].

The prevalence of poor physical function is greater in more impoverished settings, including in emerging economies [15]. Through a test that assesses impairment in gait, balance and muscle strength, women from countries with social and economic disadvantages had lower physical performance compared to women from wealthier countries [15]. It has been shown that unfavourable reproductive history such as adolescent pregnancy and multiparity are associated with poor financial and educational conditions [16] and also with worse physical performance [8, 9]. As urinary incontinence and pelvic organ prolapse are also related to pregnancy-factors [17, 18], these conditions may further explain the high rates of observed worse physical performance in poor populations.

Although there is evidence regarding the negative impact of urinary incontinence and pelvic organ prolapse on some aspects of women’s lives [13, 17], the possible relationship between these disorders and aging-related physical performance decline has rarely been explored. To the best of our knowledge, no study tested associations between these pelvic floor disorders and the physical performance of middle-aged women. The main objective of this study was to examine whether symptoms of urinary incontinence and pelvic organ prolapse are independent risk factors for reduced physical performance in middle-aged women from Northeast Brazil.

## Methods

This study took place in the Northeast of Brazil in the city of Parnamirim. This city is located in Natal’s metropolitan region, capital of Rio Grande do Norte state with a 100% urbanized population of about 200,000 inhabitants.

In this paper, we used cross-sectional data from an ongoing longitudinal research program examining physical performance in middle-aged women. The longitudinal study aims to analyze the influence of hormone levels on sarcopenia and physical functioning, and had two waves of data collection: the baseline in 2013, and the first follow-up collected between September of 2014 and July of 2015. This paper presents data collected in the first follow-up, when data related to pelvic floor disorders were collected.

### Population and sample

Five hundred women aged 40 to 65 living in Parnamirim comprised the baseline study sample [9]. The recruitment of women was performed through the dissemination of information about the project in primary health care centers around the city, where women were invited to participate. Exclusion criteria included: neurological impairments and painful conditions such as muscle and joint pain that might have compromised the physical performance measurement. In the follow-up data collection, 381 women from the cohort were re-evaluated regarding physical performance and gynecological disorders. The basic characteristics of this sample were not different from the baseline sample (data available upon request).

### Procedures

All women were assessed in a community center in Parnamirim by trained interviewers (undergraduate and graduate physiotherapy students) using standardized protocols, as described below.

#### Physical performance

Physical performance was assessed by conducting three tests: gait speed, chair stands and standing balance.

For gait speed, a 4-m walk at the subject’s usual pace was timed. The participant was asked to repeat the walk two times, and the faster of them was used to calculate gait speed (meters per second).

For the chair stand test, the participant was asked to stand up and sit down from a straight-backed chair five times, as quickly as possible, with arms crossed over the chest. This activity was timed in seconds from the initial sitting position to the fifth standing position [5].

Balance was assessed by the duration of unaided one-legged standing [6, 19]. The participant was advised to maintain a one-legged stance and the time (in seconds) from the moment the participant started the position without help until when she failed to maintain the posture was measured, up to a maximum of 30 s [6]. Two tests for each leg were performed, one with the participant’s eyes open and one with eyes closed. The mean of these two measurements was used, separately for eyes open and eyes closed and for each leg [19].

#### Urinary incontinence and pelvic organ prolapse

Urinary incontinence and symptomatic pelvic organ prolapse were self-reported as described below.

To evaluate symptoms of urinary incontinence participants were asked: “*In the last 12 months, have you lost even a small amount of urine, whether in an effort, sneezing, coughing or accidentally?*”. The possible answers were: never, less than once a month, once to several times a month, once to several times a week, every day and/or night. Those reporting no urine loss in the past year and those reporting infrequent episodes (never and less than once a month) composed the first group (reference category). Those who reported frequent episodes of urine loss (once to several times a month, once to several times a week and every day and/or night) composed the second group. Self-reported incontinence questions are acceptable research tools and widely used in epidemiologic studies, since they have moderate reproducibility and agreement with bladder diary [20–22].

To evaluate symptoms of pelvic organ prolapse, participants were asked: “*Do you have a sensation that there is a bulge in your vagina or that something is falling out from your vagina?*”. The women who answered “yes” composed one group, and those who answered “no” composed the reference group. This question is a reliable tool for the evaluation of symptoms pelvic organ prolapse since it has been reported that the sensation of a bulge or something is falling out from the vagina is 81% predictive of prolapse, and the lack of these symptoms is 76% predictive of not having pelvic organ prolapse [23].

#### Covariates

Based on previous research [9, 17, 18], the following factors were considered as potential confounders for the associations under investigation and adjusted for in multivariate analyses.

##### Age

Participant age was considered as a potential covariate, because it is associated to physical functioning [6], and to pelvic floor disorders [11, 17], according to the literature.

##### Socioeconomic position

Family income was categorized, using as a reference the Brazilian minimum monthly wage (MW), which is defined as the lowest remuneration that employers may legally pay workers. This variable was dichotomized into as ‘less than 3 minimum wage (MW), and ‘3 MW or more’. The cutoff point of 3 MW was chosen based on data from the Statistics and Socioeconomic Studies Department of Brazil, which states that the MW should be at least four times the value stablished by Brazilian government during the period of this data collection, to be able to supply the basic needs of a family in Brazil. Education was assessed as years of schooling and then categorized into: ‘less than basic education (up to seven years)’, ‘between basic and secondary (more than seven and less than eleven years)’ and ‘secondary or more (eleven years and over)’ [9]. Socioeconomic position is associated to physical functioning [15] and pelvic floor disorders [17, 18] previously.

##### Body mass index (BMI)

Obese individuals need more muscle strength to move their body mass than normal-weight persons, while their performance on activities such as gait speed and chair stand are worse [24]. Obesity may impair pelvic floor function through mechanisms such as an increase in intra-abdominal pressure and injury of pelvic musculature [25]. BMI (kg/m^2^) was calculated from height measured in meter (m) and weight measured in kilogram (kg) and then, according to the international classification from the World Health Organization (WHO) participants were categorized into three BMI groups: ‘18.5 to 24.99 (normal weight)’, ‘25.00 to 29.99 (overweight)’, ‘≥30.00 (obese)’ [26].

##### Parity

Parity was dichotomized into ‘less than three births’ and ‘three births or more’. This cut-off was selected based on evidence that having three or more children is associated to worse physical performance [9]. The number of lifetime births is also related to pelvic organ prolapse and urinary incontinence [17, 18].

##### Age at first birth

Age at first birth is related to pelvic organ prolapse, urinary incontinence, worse physical performance [9, 27, 28] and was dichotomized into “before 18 years” and “after 18 years”.

### Ethics

All participants were informed of the objectives and procedures of the research study at the time of first contact and signed a consent form, in accordance with Declaration of Helsinki and Resolution 466/12 of the National Health Council. The study protocol received ethics approval from the Ethics and Research Committee of the Federal University of Rio Grande do Norte (approval number 387.737).

### Data analysis and conceptual framework

Means and standard deviations of quantitative variables and frequencies of categorical variables were estimated in the total sample. We completed the descriptive analysis by estimating means and standard deviations of gait speed, chair stands and balance test results within categories of the independent variables and then compared them using t-tests and ANOVA test statistics.

Then, multivariate linear regression models were constructed to estimate independent effects of the pelvic floor disorders on each physical performance measure, adjusted for the potential confounders (age, education, family income, body mass index and parity). Since parity is a proxy of age at first birth [29] making both variables highly collinear, to avoid unreliable estimates as a result of collinearity in multivariate analyses, we only included the parity variable. The associations between the gynecological disorders and physical performance are essentially identical in the models adjusted by age at first birth or parity (Table 4 in Appendix).

All analyses were carried out using SPSS software, version 20.0 (SPSS, Chicago, IL, USA).

## Results

Table 1 presents the characteristics of the sample. Almost one third of all participants reported frequent episodes of urinary incontinence (at least more than once a month). The prevalence of women reporting symptomatic pelvic organ prolapse was smaller, at less than 20%.

**Table 1:** Sample characteristics (*N* = 381)

Variables	N(%) or Mean (SD)
Age (years)	51.8 (5.6)
Education	
Less than basic education	160 (42.0)
Between basic and secondary	153 (40.2)
Secondary or more	68 (17.8)
Family income	
< 3 MW	272 (71.4)
≥ 3 MW	109 (28.6)
BMI	
Normal	79 (20.7)
Overweight	156 (40.9)
Obese	146 (38.3)
Parity	
0–2 children	182 (47.8)
≥ 3 children	199 (52.2)
Age at first birth^a^	
Before 18 years old	78 (21.7)
After 18 years old	282 (78.3)
Urinary incontinence in the last 12 months	
Frequent episodes	121 (31.8)
Infrequent or none	260 (68.2)
Pelvic Organ Prolapse	
Yes	66 (17.3)
No	315 (82.7)
Chair stand (s)	10.1 (2.0)
Gait speed (m/s)	0.99 (0.17)
Balance	
Leg standing with eyes open (s)	23.2 (8.1)
Leg standing with eyes closed (s)	7.7 (6.1)

*MW* minimum wage, *BMI* body mass index

^a^parous women

Comparisons of means of dependent variables of physical performance indicators, across categories of independent variables are presented in Table 2. Bivariate analyses showed that pelvic floor complications are associated to physical performance. Women with symptoms of pelvic organ prolapse performed worse on the balance test with open eyes (*p* = 0.03). Although no statistically significant difference was observed (*p* = 0.09), a worse mean performance in the chair stand test was observed among those who reported frequent episodes of urinary incontinence.

**Table 2:** Means and standard deviations of physical performance indicators according to the categorical covariates and independent variables

	Chair stand (s)^a^	Gait speed (m/s)^b^	Balance^c^	
			Leg standing with eyes open (s)	Leg standing with eyes closed (s)
Variables	Mean (SD)			
Education				
Less than basic education	10.0 (1.8)	0.97 (0.19)	22.0 (8.6)	7.5 (6.5)
Between basic and secondary	10.0 (2.0)	1.00 (0.16)	23.8 (7.9)	7.8 (5.7)
Secondary or more	10.0 (2.5)	1.03 (0.17)	24.6 (7.3)	8.0 (5.9)
*P* value	0.97^*^	0.04^*^	0.05^*^	0.86^*^
Family income				
< 3 MW	10.0 (2.1)	0.99 (0.18)	23.0 (8.2)	7.5 (5.9)
≥ 3 MW	9,9 (1.9)	1.00 (0.16)	23.6 (8.0)	8.3 (6.5)
*P* value	0.58^**^	0,50^**^	0.57^**^	0.21^**^
BMI				
Normal	9.7 (1.8)	1.02 (0.16)	25.6 (6.2)	9.1 (6.8)
Overweight	9.9 (1.9)	1.01 (0.20)	23.5 (8.1)	7.7 (6.3)
Obese	10.2 (2.2)	0.96 (0.15)	21.6 (8.7)	7.0 (5.2)
*P* value	0.05^*^	0.001^*^	0.11^*^	0.01^*^
Parity				
0–2 children	9.9 (1.8)	1.01 (0.16)	23.9 (7.8)	8.6 (6.5)
≥ 3 children	10.1 (2.2)	0.98 (0.19)	22.5 (8.4)	6.9 (5.5)
*P* value	0.24^**^	0.11^**^	0.10^**^	0.009^**^
Age at first birth				
Before 18 years old	10.2 (2.2)	0.98 (0.19)	23.4 (7.9)	6.8 (6.1)
After 18 years old	9.8 (2.0)	1.00 (0.17)	23.0 (8.2)	7.8 (6.0)
*P* value	0.34^**^	0.42^**^	0.72^**^	0.22^**^
Urinary incontinence in the last 12 months				
Frequent episodes	10.3 (2.3)	0.98 (0.16)	23.0 (8.2)	7.6 (6.6)
Infrequent or none	9.9 (1.9)	1.00 (0.18)	23.3 (8.1)	7.8 (5.8)
*P* value	0.09^**^	0.41^**^	0.78^**^	0.83^**^
Pelvic Organ Prolapse				
Yes	9.9 (2.1)	0.98 (0.19)	21.2 (8.6)	6.6 (6.2)
No	10.0 (2.0)	0.99 (0.17)	23.6 (8.0)	7.9 (6.0)
*P* value	0.61^**^	0.64^**^	0.03^**^	0.11^**^

*MW* minimum wage, *BMI* body mass index

^a^Chair stands were evaluated by asking the participants to stand up and sit down as quickly as possible. Greater measures indicate longer times to complete the task (worse performance)

^b^Gait was measured with a four meter walk at the participants’ usual speed. Greater measures indicate faster gait speed (better performance)

^c^Balance was measured by asking the participant to stand unaided on one leg, with eyes open and closed. High values indicate better ability to sustain a position (better performance)

^*^p value for ANOVA

^**^p value for T-test

Table 3 shows the results of multivariate linear regression analysis. After adjustment for potential confounders, symptoms of urinary incontinence were significantly related to the chair stand test. Women reporting frequent episodes of urinary incontinence took on average half a second more to perform the chair stand test (β coefficient = 0.505, p = 0.03) than those reporting infrequent or no episodes. Symptoms of pelvic organ prolapse shortened the balance time with eyes open by 2.5 s on average (β coefficient = − 2.556, *p* = 0.02). The following covariates remained significantly related to physical performance indicators after statistical adjustment in the multivariate regression models: age (chair stand test, balance eyes open and closed), family income (balance eyes closed) and BMI (balance eyes open and closed and gait speed).

**Table 3:** Fully adjusted models: multiple linear regressions for the outcome variables of chair stand test, gait speed, balance eyes open, balance eyes closed

Variables	Chair stands (s)^a^			Gait speed (m/s)^b^			Balance eyes open (s)^c^			Balance eyes closed (s)^c^		
Pelvic Organ Prolapse	B	95% CI	*P* value	B	95% CI	*P* value	B	95% CI	*P* value	B	95% CI	*P* value
Yes	− 0.132	− 0.711: 0.447	0.65	− 0.015	− 0.065: 0.035	0.55	−2.556	− 4.769: − 0.343	0.02	− 1.075	−2.718: 0.567	0.19
No	0			0			0			0		
Urinary incontinence												
Frequent episodes	0.505	0.034: 0.976	0.03	− 0.015	− 0.056: 0.025	0.45	0.341	−1.454: 2.136	0.70	0.226	− 1.107: 1.559	0.73
Infrequent or none	0			0			0			0		
Age	0.043	0.002: 0.083	0.03	−0.001	− 0.004: 0.003	0.64	− 0.280	− 0.434: − 0.127	< 0.001	− 0.219	− 0.333: − 0.105	< 0.001
Family income												
< 3 MW	0.174	− 0.323: 0.671	0.49	0.004	− 0.038: 0.047	0.83	− 0.544	− 2.457: 1.368	0.57	− 1.484	− 2.908: − 0.060	0.04
≥ 3 MW	0			0			0			0		
Education												
Less than basic education	−0.325	− 1.039: 0.388	0.37	−0.046	− 0.107: 0.016	0.14	− 1.323	− 4.066: 1.420	0.34	1.846	−0.190: 3.882	0.07
Between basic and secondary	0.045	−0.616: 0.706	0.89	−0.030	− 0.087: 0.027	0.30	−0.965	−3.514: 1.585	0.45	0.591	−1.303: 2.485	0.54
Secondary or more	0			0			0			0		
BMI												
Obese	0.496	−0.111: 1.102	0.10	−0.052	−0.104: − 0.001	0.04	−4.202	−6.499: −1.905	< 0.001	− 2.336	−4.043: − 0.629	0.007
Overweight	0.194	−0.401: 0.789	0.52	−0.002	− 0.053: 0.049	0.92	−2.550	−4.810: − 0.290	0.02	−1.907	−3.584: − 0.230	0.02
Normal	0			0			0			0		
Parity												
3 or more children	0.277	−0.195: 0.750	0.24	−0.014	− 0.054: 0.027	0.51	0.121	−1.697: 1.939	0.89	−1.177	−2.530: 0.176	0.08
0–2 children	0			0			0			0		

*MW* minimum wage, *BMI* body mass index

^a^Chair stands were evaluated by asking the participants to stand up and sit down as quickly as possible. Greater measures indicate longer times to complete the task (worse performance)

^b^Gait was measured with a four meter walk at the participants’ usual speed. Greater measures indicate faster gait speed (better performance)

^c^Balance was measured by asking the participant to stand unaided on one leg, with eyes open and closed. High values indicate better ability to sustain a position (better performance)

## Discussion

This study examined the relationship between symptoms of urinary incontinence, pelvic organ prolapse and physical performance in middle-aged women. We conceptualized that these pelvic floor disorders are potential risk factors for decline in the physical performance of middle-aged women.

Concerning the prevalence of these disorders, we found that 31.8% of women in our study complained of frequent episodes of urinary incontinence and 17.3% from symptoms of pelvic organ prolapse. Previous studies reported different proportions of self-reported urinary incontinence in middle-aged women, varying from 23.6% in a Brazilian population [30] to 37.2% in Chinese women [31]. These differences may be attributed to the differences in the questionnaires used to evaluate and to the population under study. The prevalence of symptoms of pelvic organ prolapse is even wider among the different studies and populations, varying from smaller proportions in studies from high income populations, at 3.8 and 2.1% in middle-aged women from US [32, 33], to greater proportions, similar to ours, in developing countries, at 16.2 and 18.0% for middle-aged women from Bangladesh and Lebanon, respectively [34, 35].

The results of our multivariate regression models suggest that women with symptoms of urinary incontinence and pelvic organ prolapse have worse physical performance during the chair stand and balance with eyes open tests, respectively. Different stages of the women’s reproductive history, such as pregnancy, the postpartum period and menopause can impose stress on pelvic floor muscles [10], which may result in urinary incontinence and pelvic organ prolapse. It is suggested that these conditions are caused by direct mechanical damage to fascia, ligaments, peripheral nerves and muscles that support pelvic organs and are essential for maintaining continence [10].

Women with dysfunction of the pelvic floor, such as urinary incontinence, tend to avoid risks and constraints, because of the fear of losing urine publically [13]. This may explain why they perform chair stand tests slower; that is, they are concerned that the test itself may cause an involuntary loss of urine. Fritel and colleagues [36] found similar results in a sample of 1942 French older women. These authors also reported that the severity of urinary incontinence plays an important role on the performance of simple functional tests, since those reporting severe urinary incontinence presented even worse results of performance on motor and balance skills than those reporting lower degrees [36]. Furthermore, a previous study by Corrêa and colleagues [14] found that self-reported UI was associated with worse and more pronounced declines in physical performance over two years in a study of 915 community-dwelling older women.

There is also a possibility of reverse causality in this cross-sectional analysis. It seems that some physical function tests are associated with the development of urinary incontinence later on, but there are discrepancies in the literature. In one longitudinal study, women with lower scores on the chair stand were more likely to develop urinary incontinence in the subsequent three years [37]. The authors affirmed that could be due to the difficulty getting to the bathroom or multi-factorial causes of both immobility and incontinence [37]. Similarly, Jerez-Roig and colleagues [38] found that disability and functional decline are significant predictors of urinary continence decline after two years in a sample of institutionalized older adults. However, Krause et al. (2010) [39] showed that 6-min walk test, chair stand test, and test of sit-and-reach were not significant predictors of urinary incontinence [39]. More longitudinal studies are needed to shed light on this complex association and clarify the cause and effect relationships.

We also observed that women who suffer from symptoms of pelvic organ prolapse exhibit worse physical performance in balance test with eyes open. As reported previously [40], a possible explanation for this finding could be the physical discomfort felt by these women during the single leg stance, leading to shorter test performance time. Postural stability generally depends on interactions between various neural, visual, and vestibular structures. During the balance test with eyes closed, the vestibular system is essential to maintain the single leg stance. However, for the balance test with eyes open, in addition to the required coordinated contraction of muscle to stabilize the hip, pelvis and lower limb, the visual system also plays a pivotal role [18]. Given that the weakness of pelvic floor muscles is the main cause of symptomatic pelvic organ prolapse [11], a more pronounced relationship between symptomatic pelvic organ prolapse and the balance with eyes open (compared to the balance with eyes closed) is not unexpected. There is no documented ill effect on the vestibular system (the main determinant of balance with eyes closed) from pelvic floor disorders.

Although the differences between the groups of urinary incontinence and pelvic organ prolapse may seem small, it has been reported that small changes on physical performance are clinically relevant [41] and increase mortality and mobility disability risk among older adults [42]. For instance, small differences as of 0.3 and 0.5 points in a battery that evaluates the lower limbs physical functioning, the SPPB (Short Physical Performance Battery), can be considered a meaningful change in older adults [43, 44]. The SPPB evaluates lower limbs physical performance by tests of balance, gait speed and chair stands, with scores varying from 0 to 12. Although there are no studies evaluating the minimal clinically important changes for the specific performance tests used in this study, it has been reported that an increase of only 1 s in the time to perform the chair stands increases the likelihood of older women having sarcopenia (loss of muscle mass) by 8% [41].

We also identified other significant determinants for the physical functioning in our sample. Consistent with other studies [23, 45], we found a significant association between higher BMI and gait speed, balance with eyes open and balance with eyes closed. A higher amount of adipose tissue is associated with accumulation of intramuscular fat, a decrease of muscle quality and thus poor muscular function [46]. Also as expected, there were significant associations between age and the chair stand, balance with eyes open and closed. Our findings are supported by literature examining aging-related musculoskeletal changes, in that the time needed to complete the chair stand tests increases [47], and the performance time for the eyes open and closed balance tests becomes shorter [18].

Although urinary incontinence does not pose a threat to life, it has a negative influence on the physical, psychological and social condition of women and is associated with a significant decrease in the quality of life and increased health care costs [48]. A previous study suggested that women with urinary incontinence, especially those living in the community, are at higher risk of falls, generally do not seek medical treatment, and do not perceive their condition as a major health problem [46]. These authors [49] showed that women with nocturnal urine loss were at higher risk for falls and concurrently had poorer scores on physical performance tests of balance, gait speed and chair stand [49]. Fall is a common occurrence in older adults with considerable public health burdens and understanding factors predicting physical performance will have important social and public policy implications. Development of specific interventions for management of pelvic floor disorders in vulnerable populations is one strategy for prevention of future physical disabilities.

There are few studies examining relationships between pelvic floor disorders and physical performance, and to the best of our knowledge, no such study has been conducted among Brazilian middle-aged women. Similar studies are needed to further investigate the relationship between urogynecological disorders and physical performance in different settings.

This study has some limitations. Lack of temporality due to cross-sectional design limits causal inference and we are not certain whether the measured pelvic floor conditions impact physical performance or vice versa. Longitudinal studies are needed for clarification. In addition, it was not possible to perform a clinical evaluation of these urogynecological disorders. There is a possibility of under-reporting of these conditions because of the self-report method of assessment of urinary incontinence and pelvic organ prolapse. However, there is no reason to think people with poor physical function under report these conditions differently, and any associated misclassification would more likely be non-differential in nature, most typically leading bias towards no effect. Moreover, our small sample size did not allow us to examine the total range of frequencies of urinary incontinence and all categories were collapsed into two groups and the variable we analyzed as a dichotomous variable. It is possible that different frequencies in the urinary incontinence episodes would be associated differently with physical performance measures, as described previously [35]. Another possible limitation was the fact that respiratory and cardiovascular diseases were not considerate as exclusion criteria. However, the prevalence of these diseases were low in our sample (4 and 5%, respectively, for respiratory and cardiovascular condition) thus, it’s unlikely that these conditions could confound our relationship of interest.

Despite these limitations this study is important because of its novel research question and approach. The few studies that investigated associations between urogynecological conditions of urinary incontinence and physical performance [12, 49] were mainly in older adult populations. Although the differences between the groups were relatively small, similar small changes in physical performance increase mortality and mobility disability risk among older adults [42] and are considered meaningful changes in older adults [43, 44]. As far as we know, there are no studies evaluating the minimal clinically important differences for the specific tests of physical performance we used in our study, but we believe that if women presenting urogynecological disorders are already presenting worse physical functioning in middle-age, and they may have higher rates of mobility disabilities and morbidities at older ages. Thus, this study with a focus on middle-aged women may contribute to better understanding of the mechanisms related to changes in physical performance at younger ages. Furthermore, this study could serve as a basis for further research in order to improve urogynecological function and consequently help prevent early decline in physical functioning and therefore enhance women’s quality of life.

## Conclusion

This study aimed to analyze associations between symptoms of urinary incontinence, pelvic organ prolapse and physical performance in middle-aged women. Women with symptoms of pelvic organ prolapse had worse physical performance in balance test with eyes open and those with symptoms of urinary incontinence had worse chair stand test results.

There is a need for further research on these urogynecological conditions and the implementation of preventive interventions to improve women’s aging profile and prevent disability in women in older ages. In addition, this study provides important information for targeted preventive behaviors, health promotion measures, and treatment options for middle-aged women to be used by policy makers and clinicians such as physiotherapists and gynecologists.

## Data Availability

The datasets used and/or analyzed during the current study are available from the corresponding author on reasonable request.

## Appendix

**Table 4:** Fully adjusted models: multiple linear regressions for the outcome variables of chair stand test, gait speed, balance eyes open, balance eyes closed, with further adjustment for age at first birth

Variables	Chair stands (s)^a^			Gait speed (m/s)^b^			Balance eyes open (s)^c^			Balance eyes closed (s)^c^		
Pelvic Organ Prolapse	B	95% CI	*P* value	B	95% CI	*P* value	B	95% CI	*P* value	B	95% CI	*P* value
Yes	−0.118	− 0.697: 0.461	0.68	−0.016	− 0.066: 0.034	0.52	− 2.579	−4.787: − 0.372	0.02	− 1.119	−2.763: 0.524	0.18
No	0			0			0			0		
Urinary incontinence												
Frequent episodes	0.502	0.030: 0.973	0.03	−0.015	− 0.056: 0.025	0.44	0.327	− 1.466: 2.120	0.72	0.234	−1.101: 1.559	0.73
Infrequent or none	0			0			0			0		
Age	0.048	0.007: 0.088	0.02	−0.001	−0.004: 0.002	0.58	−0.272	− 0.424: − 0.119	< 0.001	−0.240	− 0.354: − 0.127	< 0.001
Family income												
< 3 MW	0.157	−0.342: 0.656	0.53	0.004	−0.039: 0.047	0.85	−0.629	−2.547: 1.289	0.51	− 1.431	−2.863: 0.000	0.05
≥ 3 MW	0			0			0			0		
Education												
Less than basic education	−0.271	−0.968: 0.426	0.44	−0.051	− 0.111: 0.009	0.09	−1.525	−4.200: 1.151	0.26	1.605	−0.387: 3.597	0.11
Between basic and secondary	0.069	−0.591: 0.728	0.83	−0.032	− 0.089: 0.025	0.27	−0.998	−3.535: 1.539	0.44	0.492	−1.399: 2.382	0.60
Secondary or more	0			0			0			0		
BMI												
Obese	0.495	−0.112: 1.102	0.11	−0.053	−0.104: − 0.001	0.04	−4.252	−6.546: −1.958	< 0.001	−2.348	−4.058: − 0.638	0.007
Overweight	0.209	−0.387: 0.804	0.49	−0.002	− 0.053: 0.049	0.93	−2.550	−4.759: − 0.241	0.03	−1.973	− 3.655: − 0.292	0.02
Normal	0			0			0			0		
Age at first birth												
Before 18 years old	0.271	−0.282: 0.824	0.33	0.000	− 0.047: 0.047	0.98	1.046	−1.059: 3.150	0.32	−1.081	−2.647: 0.486	0.17
After 18 years old	0			0			0			0		

*MW* minimum wage, *BMI* body mass index

^a^Chair stands were evaluated by asking the participants to stand up and sit down as quickly as possible. Greater measures indicate longer times to complete the task (worse performance)

^b^Gait was measured with a four meter walk at the participants’ usual speed. Greater measures indicate faster gait speed (better performance)

^c^Balance was measured by asking the participant to stand unaided on one leg, with eyes open and closed. High values indicate better ability to sustain a position (better performance)

## Abbreviations

BMI: Body Mass Index
MW: Brazilian minimum monthly wage
